# Supplementary material for: Developmental, cellular, and behavioral phenotypes in a mouse model of congenital hypoplasia of the dentate gyrus
Source: eLife. 2020 Oct 21;9:e62766. doi: 10.7554/eLife.62766 (PMC7577738; doi:10.7554/eLife.62766)
Supplement: Supplementary file 1. — Mouse identification numbers are indicated beneath the genotype. [file elife-62766-supp1.docx]

Supplementary file 1. Sampling parameters chosen for the Optical Fractionator Probe for two hemispheres. Mouse identification numbers are indicated beneath the genotype.

|  | *Wls^fl/+^;Gfap-Cre* (4931) | *Wls^fl/-^;Gfap-Cre* (4908) |
| --- | --- | --- |
| Number of sections counted | 25 | 26 |
| Number of missing sections | 4 | 0 |
| Section interval | 1 | 1 |
| Sampling grid | 65x65 um | |
| Counting frame | 50x50 um | |
| Dissector height | 70 m | |
| Guard Zone | 5 % | |
