# Supplementary material for: Developmental, cellular, and behavioral phenotypes in a mouse model of congenital hypoplasia of the dentate gyrus
Source: eLife. 2020 Oct 21;9:e62766. doi: 10.7554/eLife.62766 (PMC7577738; doi:10.7554/eLife.62766)
Supplement: Supplementary file 2. — Mouse identification numbers and left hemisphere (LH) designation are indicated beneath the genotype. [file elife-62766-supp2.docx]

Supplementary file 2. Area sampling outcomes of the Optical Fractionator Probe for two hemispheres. Mouse identification numbers and left hemisphere (LH) designation are indicated beneath the genotype.

|  | *Wls^fl/+^;Gfap-Cre* | *Wls^fl/-^;Gfap-Cre* |
| --- | --- | --- |
|  | (4931 LH) | (4908 LH) |
| ***Estimated volume (µm3)*** | 5738854000 | 513914000 |
| ***Area sampled(µm2)*** | 4046620 | 4282620 |
| ***Missing sections*** | 4 | 0 |
| ***Total thickness weighted*** | 90610,5 | 53516,9 |
| ***Thickness sites with counts*** | 86,95 | 80,12 |
| ***Sites with counts*** | 369/1415 (26 %) | 329/1433 (22 %) |
