# Supplementary material for: Developmental, cellular, and behavioral phenotypes in a mouse model of congenital hypoplasia of the dentate gyrus
Source: eLife. 2020 Oct 21;9:e62766. doi: 10.7554/eLife.62766 (PMC7577738; doi:10.7554/eLife.62766)
Supplement: Supplementary file 3. — Using an optical fractionator probe, complex spines were classified and counted in the left hemisphere of Wlsfl/+;Gfap-Cre and Wlsfl/-;Gfap-Cre mice. The table shows the number of counted spines and, in parentheses, stereologically estimated spines. Five distinct complex spine subtypes were classified: basic, big/prototypical, long, thin, and tall. [file elife-62766-supp3.docx]

Supplementary file 3. Stereological analyses of complex spines in CA3. Using an Optical Fractionator probe, complex spines were classified and counted in the left hemisphere of *Wls^fl/+^;Gfap-Cre* and *Wls^fl/-^;Gfap-Cre* mice. The table shows the number of counted spines and, in parentheses, stereologically estimated spines. Five distinct complex spine subtypes were classified: basic, big/prototypical, long, thin, and tall.

|  | *Wls^fl/+^;Gfap-Cre* | *Wls^fl/-^;Gfap-Cre* |
| --- | --- | --- |
|  | (4931 LH)  count (estimate) | (4908 LH)  count (estimate) |
| ***Basic spines*** | 453 (1115) | 212 (408) |
| *Gundersen Coefficient of Error* | 0,05 | 0,07 |
| ***Big/prototypical spines*** | 107 (263) | 31 (59) |
| *Gundersen Coefficient of Error* | 0,1 | 0,18 |
| ***Long spines*** | 178 (438) | 167 (321) |
| *Gundersen Coefficient of Error* | 0,08 | 0,08 |
| ***Tall spines*** | 209 (514) | 180 (346) |
| *Gundersen Coefficient of Error* | 0,07 | 0,07 |
| ***Thin spines*** | 99 (246) | 74 (142) |
| *Gundersen Coefficient of Error* | 0,1 | 0,12 |
| ***Total spines*** | 1046 (2576) | 664 (1292) |
| *Gundersen Coefficient of Error* | 0,03 | 0,04 |
