# Supplementary material for: Developmental, cellular, and behavioral phenotypes in a mouse model of congenital hypoplasia of the dentate gyrus
Source: eLife. 2020 Oct 21;9:e62766. doi: 10.7554/eLife.62766 (PMC7577738; doi:10.7554/eLife.62766)
Supplement: Supplementary file 4. — The data shown here were collected on the cohort of mice tested at New York University (Figures 6 and 7). N = 15 Wlsfl/+;Gfap-Cre mice; N = 14 Wlsfl/-;Gfap-Cre mice. [file elife-62766-supp4.docx]

Supplementary file 4. Baseline neurological tests and their outcomes. The data shown here were collected on the cohort of mice tested at New York University (Figures 6 and 7). N=15 *Wls^fl/+^;Gfap-Cre* mice; N=14 *Wls^fl/-^;Gfap-Cre* mice.

| **Test** | **Outcome**  [measure] | *Wls^fl/+^;Gfap-Cre*  avg ± s.e.m. | *Wls^fl/-^;Gfap-Cre*  avg ± s.e.m. | **t value**  (df = 28) | **p** |
| --- | --- | --- | --- | --- | --- |
| Vertical screen | Climb up in 30s [0,1] | 1 ± 0.0 | 0.93 ± 0.07 | 0.96 | 0.34 |
| Spontaneous grooming | Time grooming in 5 min [s] | 17.7 ± 5.1 | 37.2 ± 12.3 | 1.46 | 0.15 |
| Elicited grooming | Time grooming in 5 min [s] | 158.0 ± 13.9 | 196.0 ± 14.1 | 1.92 | 0.07 |
| Parallel bars | Time to turn 90° [s] | 15.71 ± 3.0 | 14.8 ± 2.3 | 0.24 | 0.81 |
| Visual placing response | 3 trials  [0,1] | 3.0 ± 0 | 3.0 ± 0 | 0.0 | 1.0 |
| Negative geotaxis | Time to turn up  [s] | 14.1 ± 2.1 | 24.1 ± 1.4 | 2.02 | 0.05 |
| Suspension test | Time to drop  [s] | 26.5 ± 2.3 | 28.8 ± 1.2 | 0.90 | 0.38 |
